# Cardiopulmonary Bypass Video Is an Effective Learning Tool

**DOI:** 10.31486/toj.19.0103

**Published:** 2020

**Authors:** Michael T. Tseng, P. Eugene Parrino, G. Dodd Denton

**Affiliations:** ^1^ The University of Queensland Faculty of Medicine, Ochsner Clinical School, New Orleans, LA; ^2^ Department of Cardiovascular Surgery, Ochsner Clinic Foundation, New Orleans, LA; ^3^ Department of Internal Medicine, Ochsner Clinic Foundation, New Orleans, LA

**Keywords:** *Bypass–cardiopulmonary*, *instructional film and video*, *learning*, *schools–medical*

## Abstract

**Background:** Surgical procedures require the collaboration of medical personnel with multiple skill sets who have different levels of training. Someone new to surgical procedures, such as a medical student, faces a steep learning curve. Studies have shown that video-assisted learning is associated with improved learning of surgical procedures.

**Methods:** During their surgical rotation orientation, third-year medical students were invited via email to participate in a learning study featuring a cardiopulmonary bypass video. Study participants took a pretest, reviewed the locally developed video, and took a posttest and an attitudinal questionnaire after viewing the video.

**Results:** A convenience sample of 31 third-year medical students participated in the study. Overall knowledge scores improved from pretest to posttest (36.9% vs 79.6%, *P*<0.001). In the posttest attitudinal questionnaire, students reported that they preferred video-assisted learning to reading written protocols (90.3% strongly agree/agree) and that they were more knowledgeable about the function of the cardiopulmonary bypass machine (80.7% strongly agree/agree) after viewing the video. Students also reported that the video would be useful during their surgical clerkships (90.4% strongly agree/agree).

**Conclusion:** Video-assisted learning was associated with comprehension of the material immediately after viewing the video, and medical students considered it to be appropriate and useful. This educational video may benefit other learners who are entering the cardiopulmonary bypass operating room for the first time.

## INTRODUCTION

Entering the operating room (OR) for the first time can be overwhelming. To help prepare students and new staff for the experience, an educational video was produced at Ochsner Health to introduce cardiopulmonary bypass surgery and familiarize learners with the OR. The video introduction is appropriate for any personnel new to the OR, such as medical technologists, nursing students, medical students, and residents.

Complex surgical procedures such as cardiothoracic surgeries require all personnel in the OR to have the awareness and requisite surgical knowledge to engage and to decrease the potential for human error.^1^ Surgical teams may struggle to teach medical students in the short time frame of an operation; Sweeney suggests pairing traditional lectures with new methods of active learning such as medical simulation and video review when appropriate.^2^

Many of today's learners prefer digital and nontraditional educational methods. Studies published in 2013 and 2017 demonstrated that instructional videos provided better preparation for procedures among residents than traditional methods did, especially for procedures that were new to them.^3,4^ A 2016 study showed that instructional videos of interventional breast procedures led to efficient learning and enhanced comprehension among radiology residents.^5^ In response to a survey conducted by Hayden et al, general surgery residents in cardiac and thoracic services reported the need for easily accessible resources about surgical procedures, and that response drove the development of a surgical educational video.^6^ Video-assisted debriefing has been shown to improve learner experience, attitudes, and performance compared to verbal debriefing.^7^

The primary aims of this study were to determine if a video could improve medical students’ knowledge and understanding of cardiopulmonary bypass surgery and whether the video would be acceptable to medical students.

## METHODS

This study was reviewed and approved as exempt by the Ochsner Clinic Foundation Institutional Review Board.

**Figure 1.**
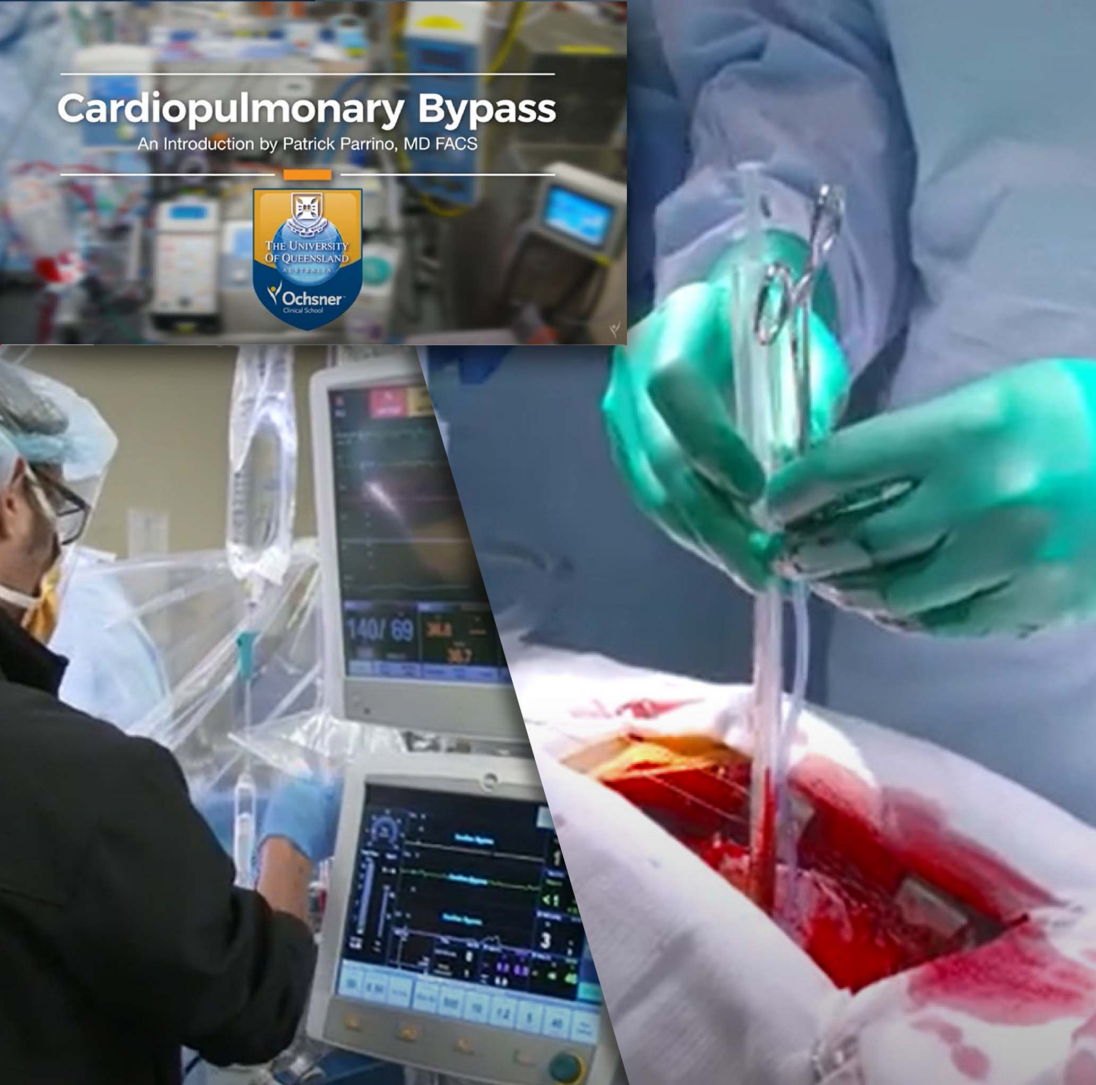
**Screen captures from the video “Cardiopulmonary Bypass – An Introduction by Patrick Parrino, MD, FACS.” Access the video at https://youtu.be/_BFoLOUXYcE.** *Warning: The video contains graphic content depicting surgery for educational purposes and may not be suitable for all viewers.*

**Figure 2.**
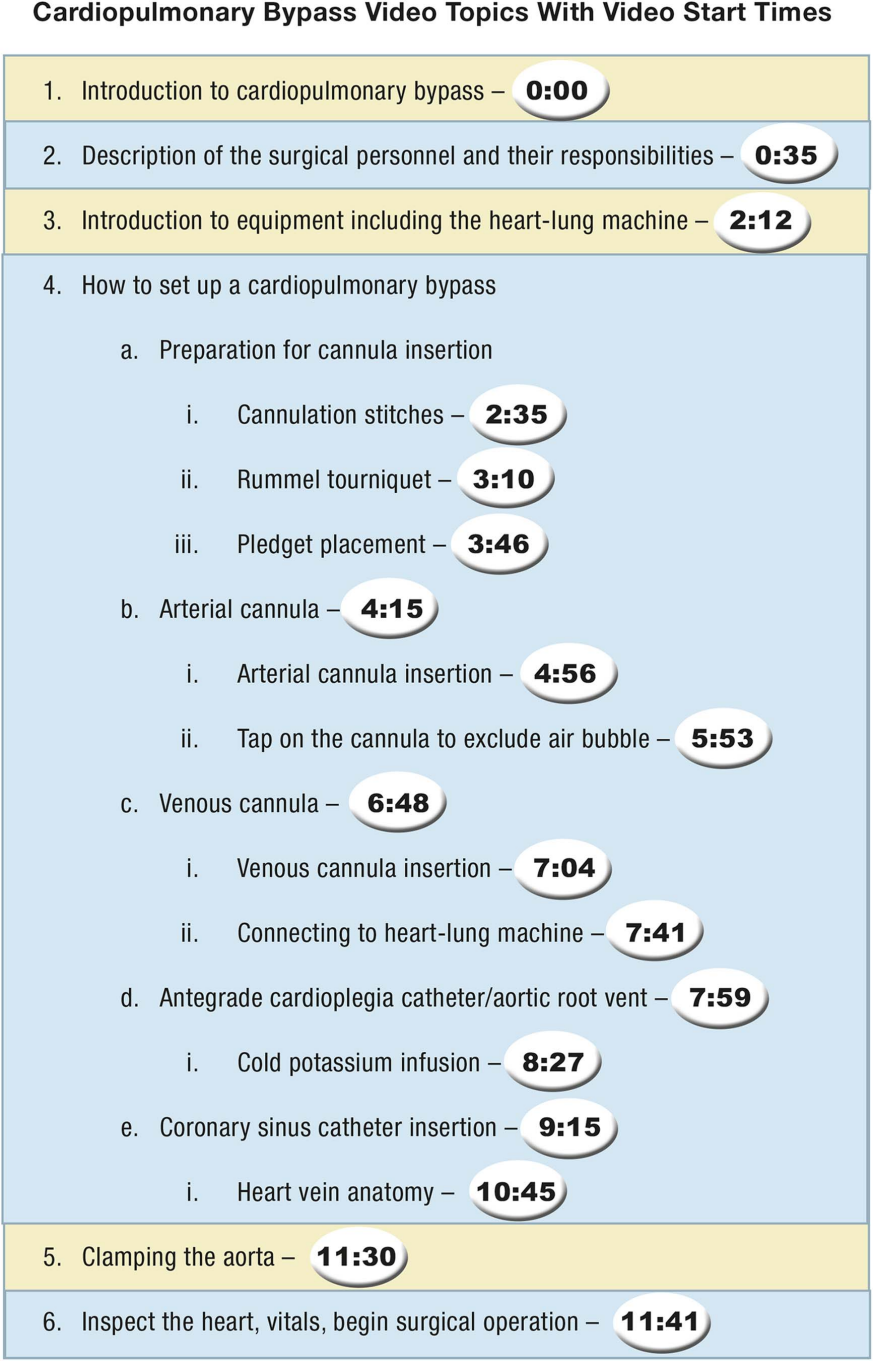
**Cardiopulmonary bypass video outline.**

One of the authors (P.E.P.) developed the video (Figure 1) prior to the study based on general observations that new personnel entering the cardiopulmonary bypass surgical suite were not well prepared. The Ochsner Clinic Foundation audiovisual department edited and produced the video. Figure 2 outlines the topics included in the video and lists the start time for each topic.

**Figure 3.**
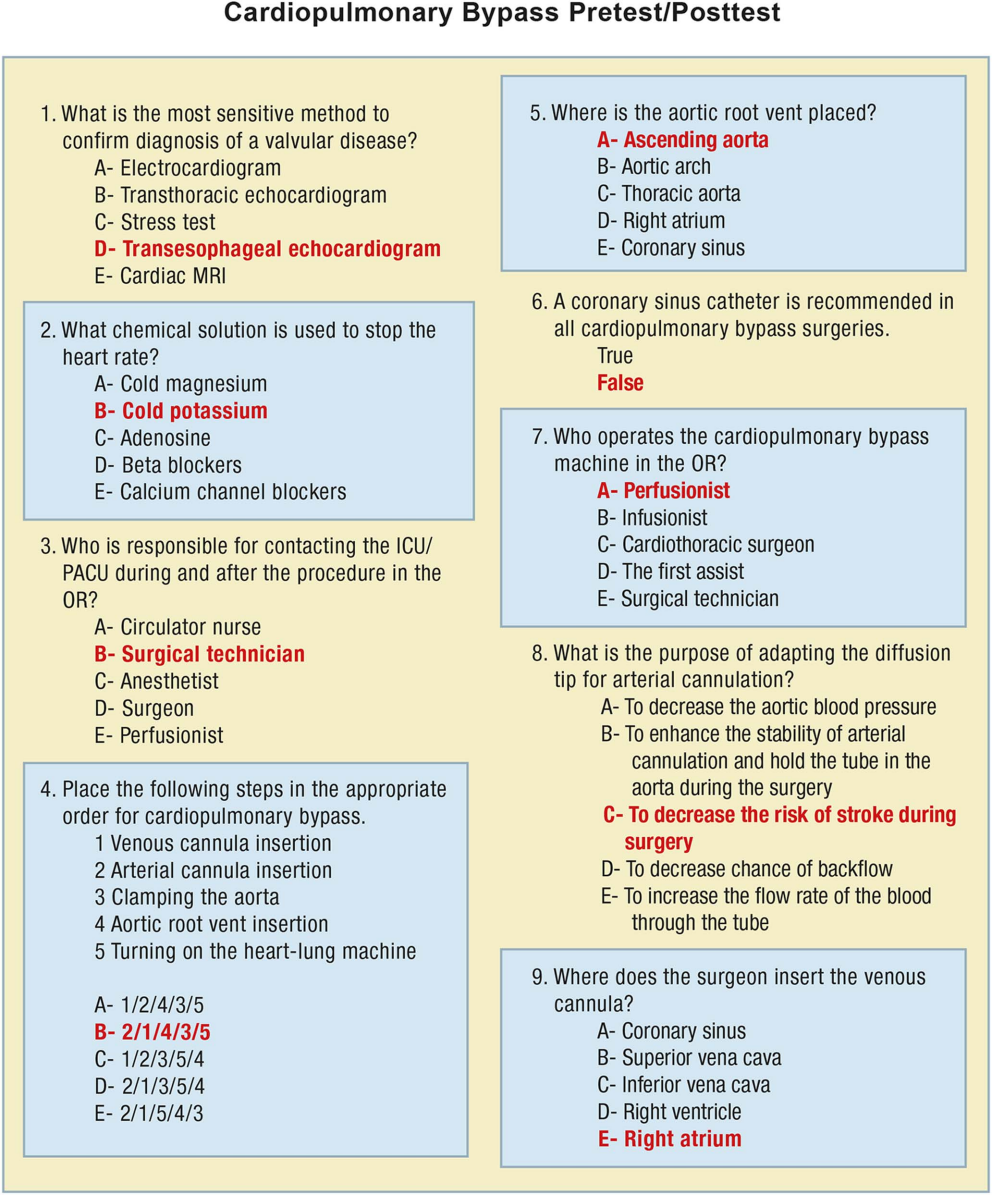
**Cardiopulmonary bypass video pretest/posttest with correct answers in boldface.** ICU, intensive care unit; MRI, magnetic resonance imaging; OR, operating room; PACU, postanesthesia care unit.

Because of positive responses from viewers and faculty members, a project to test the effectiveness of the video and disseminate it outside Ochsner Health was initiated. Two authors (M.T.T. and G.D.D.) collaborated to develop a 9-question pretest/posttest (Figure 3) to explore learner knowledge of cardiopulmonary bypass surgery and a 5-point Likert-scale questionnaire to be completed after viewing the video that explored learners’ confidence in their ability to function in the cardiopulmonary bypass surgical suite.

During their surgical rotation orientation, 54 third-year medical students from The University of Queensland-Ochsner Clinical School were invited via email to participate in this study. Volunteer participants completed the pretest, viewed the video, and completed the posttest and questionnaire.

Responses were collected and analyzed. Chi-square test was used to compare the percent correct answers before and after viewing the video. The 5-point Likert scale responses were collapsed into 3 categories (strongly agree/agree, neutral, and strongly disagree/disagree) for ease of analysis. Chi-square test was used to compare agree/strongly agree responses to disagree/strongly disagree responses.

## RESULTS

Thirty-one third-year medical students (57.4% of invited students) completed all 3 components of the assignment: pretest, video viewing, and completion of the posttest and questionnaire. As shown in Table 1, knowledge scores improved for all but one question (question 5, Where is the aortic root vent placed?) from pretest to posttest. The overall percentage of correct answers also improved (36.9% vs 79.6%, *P*<0.001).

**Table 1.** Cardiopulmonary Bypass Procedures Video Pretest and Posttest Results (n=31)

	Percentage of Students Answering Correctly		
		
Question	Pretest	Posttest	*P* value
1. What is the most sensitive method to confirm diagnosis of a valvular disease?	61.3	100	<0.001
2. What chemical solution is used to stop the heart rate?	32.3	90.3	<0.001
3. Who is responsible for contacting the ICU/PACU during and after the procedure in the OR?	29.0	80.6	<0.001
4. Place the following steps in the appropriate order for cardiopulmonary bypass.	6.5	41.9	<0.001
5. Where is the aortic root vent placed?	67.7	67.7	NS
6. A coronary sinus catheter is recommended in all cardiopulmonary bypass surgeries.	41.9	71.0	<0.001
7. Who operates the cardiopulmonary bypass machine in the OR?	64.5	96.8	<0.001
8. What is the purpose of adapting the diffusion tip for arterial cannulation?	16.1	90.3	<0.001
9. Where does the surgeon insert the venous cannula?	12.9	77.4	<0.001

Note: Chi-square test was used to compare the percent correct answers before and after viewing the video.

ICU, intensive care unit; NS, not significant; OR, operating room; PACU, postanesthesia care unit.

In the questionnaire administered after the posttest, students reported that the video content was appropriate and useful. They reported that they preferred watching the video to reading a protocol and that the video was well prepared. Students’ confidence in their knowledge of the roles of the perfusionist and circulator nurse and the function of the bypass machine increased, but they did not report increased confidence in reciting the steps of the procedure or in being ready to assist in the procedure (Table 2).

**Table 2.** Attitudinal Questionnaire Results (n=31)

Question	Strongly Agree/Agree, **%**	Neutral, **%**	Strongly Disagree/Disagree, **%**	*P* value
I am confident in reciting the steps of the cardiopulmonary bypass procedure.	35.5	38.7	25.8	NS
I am confident that I can assist in a cardiopulmonary bypass procedure.	25.8	48.4	25.8	NS
I understand the roles of the perfusionist in cardiopulmonary bypass surgery.	83.8	12.9	3.2	<0.001
I understand the roles of the circulator nurse in cardiopulmonary bypass.	93.6	6.5	0	<0.001
I am knowledgeable about how the cardiopulmonary machine functions.	80.7	9.7	9.7	<0.001
The video content is appropriate to medical students.	100	0	0	<0.001
This video was well prepared.	100	0	0	<0.001
This video will be useful to me during my surgical clerkship.	90.4	9.7	0	<0.001
I prefer watching the video over reading a written protocol.	90.3	6.5	3.2	<0.001

Notes: The 5-point Likert scale responses were collapsed into the 3 categories shown for ease of analysis. Chi-square test was used to compare the percent strongly agree/agree to strongly disagree/disagree.

NS, not significant.

## DISCUSSION

Our results aligned with the expectation that student scores on a knowledge test would increase after viewing the video. Some learners prefer video-assisted learning,^3,4,7^ and the use of video was effective in this study. In the questionnaire responses, almost all learners reported that they understood the roles in the cardiothoracic OR after viewing the video and affirmed the appropriateness and usefulness of the video. However, students did not report high confidence regarding assisting in or reciting the steps of the procedure after viewing the video.

High confidence would have been a surprising result, as cardiopulmonary bypass is among the most complex surgeries in medicine. Many years of postgraduate training are required to achieve competence. A video alone cannot be expected to increase confidence significantly; only repeated practice over time should have that outcome.

Video-assisted learning has a place in medical education,^3-7^ particularly for learners who prefer videos and for material suited for video-assisted learning, such as procedures. Comparisons of video-assisted learning and other forms of learning, such as didactics, paper cases, and simulation, have not consistently shown that students prefer one form to another.^8^

Our investigation has several limitations. While student knowledge improved, we did not have a comparison group to show if the improvement in the video group was better than the improvement demonstrated by students who reviewed a paper case or read the information in an article. A convenience sample may not be representative of the class as a whole and may contain students with more or less confidence in surgical procedures. Also, posttest results can be influenced by pretest questions. After taking a pretest, students may view the video in search of answers to the questions. To further assess learning outcomes, we may consider incorporating questions that test students’ ability to apply, recall, and synthesize information.

A future direction for study is to include a comparison group with learning presented in a different format, such as a paper case, to establish whether the video results in better knowledge outcomes. We could also attempt to recruit an entire class of students to reduce the selection bias inherent in a convenience sample. This educational video has been added to the surgical orientation curriculum.

## CONCLUSION

Entering the OR for the first time can be a daunting experience, particularly for medical students. The preclinical years of medical school education often do not prepare medical students to face the kinesthetic experiences and fast pace of the OR. Studies have shown that video-assisted learning has become a popular educational modality, and our video was effective in increasing students’ knowledge of cardiopulmonary bypass surgery.

## Notes

This article meets the Accreditation Council for Graduate Medical Education and the American Board of Medical Specialties Maintenance of Certification competencies for Patient Care, Medical Knowledge, and Practice-Based Learning and Improvement.
